# Reduced alpha diversity of the oral microbiome correlates with short progression‐free survival in patients with relapsed/refractory multiple myeloma treated with ixazomib‐based therapy (AGMT MM 1, phase II trial)

**DOI:** 10.1002/jha2.130

**Published:** 2020-11-08

**Authors:** Heinz Ludwig, Bela Hausmann, Martin Schreder, Wolfram Pönisch, Niklas Zojer, Stefan Knop, Eberhard Gunsilius, Alexander Egle, Andreas Petzer, Hermann Einsele, Roman Hajek, Katja Weisel, Karl Jochen Krenosz, Alois Lang, Daniel Lechner, Richard Greil, David Berry

**Affiliations:** ^1^ Wilhelminen Cancer Research Institute Vienna Austria; ^2^ Joint Microbiome Facility (JMF) Medical University of Vienna & University of Vienna Vienna Austria; ^3^ Department of Laboratory Medicine Medical University of Vienna Vienna Austria; ^4^ Division of Hematology and Medical Oncology Department of Internal Medicine II Würzburg University Medical Center Würzburg Germany; ^5^ Department of Hematology University of Leipzig Leipzig Germany; ^6^ Department of Medicine I Wilhelminenspital Vienna Austria; ^7^ Department of Internal Medicine V Medical University Innsbruck Innsbruck Austria; ^8^ Department of Internal Medicine III with Haematology Medical Oncology, Hemostaseology Infectiology and Rheumatology Oncologic Center Salzburg Cancer Research Institute ‐ Laboratory for Immunological and Molecular Cancer Research (SCRI‐LIMCR) Paracelsus Medical University Cancer Cluster Salzburg Salzburg Austria; ^9^ Department of Internal Medicine I Ordensklinikum Linz BHS‐EKH, Linz Linz Austria; ^10^ Department of Hematooncology University Hospital Ostrava Ostrava Czech Republic; ^11^ University of Tübingen Tübingen Germany; ^12^ Department of Internal Medicine 3 Kepler Universitätsklinikum GmbH Med Campus III Linz Austria; ^13^ Innere Medizin II LKH Feldkirch Feldkirch Austria; ^14^ Department of Medicine I ‐ Hematology with Stem Cell Transplantation Hemostaseology and Medical Oncology Ordensklinikum Linz Elisabethinen Linz Austria; ^15^ Division of Microbial Ecology Department of Microbiology and Ecosystem Science Centre for Microbiology and Environmental Systems Science University of Vienna Vienna Austria

**Keywords:** molecular analysis, mucosal, multiple myeloma

## Abstract

Alterations in the human microbiome have been linked to several malignant diseases. Here, we investigated the oral microbiome of 79 patients with relapsed/refractory multiple myeloma (MM) treated with ixazomib‐thalidomide‐dexamethasone. Increased alpha diversity (Shannon index) at the phylum level was associated with longer progression‐free survival (PFS) (10.2 vs 8.5 months, *P* = .04), particularly in patients with very long (>75% quartile) PFS . Additionally, alpha diversity was lower in patients with progressive disease (*P* < .05). These findings suggest an interrelationship between the oral microbiome and outcome in patients with MM and encourage a novel direction for diagnostic and/or therapeutic strategies.

## 1 INTRODUCTION

A stable host‐microbiome equilibrium is essential for optimal health. Recent evidence underlines the importance of a diverse and “healthy” gastrointestinal microbiome for tumor progression [1], treatment response [2], chemotherapy‐related blood stream infection [3], and survival [2]. Though the gastrointestinal microbiome has been linked with outcome in multiple myeloma [4, 5], associations between the oral microbiome and risk factors and/or outcome are lacking. Here, we evaluated whether the oral microbiome is associated with patient characteristics and treatment outcome in patients with relapsed/refractory multiple myeloma (RRMM) enrolled in a phase 2 trial with ixazomib‐thalidomide‐dexamethasone.

## 2 METHODS

Ninety patients with RRMM were enrolled in this phase II trial. Mouth swabs were available for 79 patients (median age: 67 (45‐84) years, ISS (International Staging System) stage I: 32, II 27, III: 20, median number of prior TX lines: 1 (range: 1‐6). No patient had an infection at baseline, and none had received antibiotics for 4 weeks before enrollment. Mouths swabs were obtained using 4N6FLOQSwabs™ (Thermo Fisher, Waltham, MA, USA) and stored at –20^○^C within 2 hours of collection. Total DNA was extracted using the QIAamp DNA Mini Kit (Qiagen, Hilden, Germany) [6]. The V3‐V4 region of bacterial 16S rRNA gene was amplified using 341F and 785R primers, and barcoded amplicons were sequenced on an Illumina MiSEquation (Illumina, San Diego, CA, USA). Sequences were demultiplexed and amplicon sequence variants (ASVs) were inferred using the DADA2 R package [7] and taxonomically classified using the SILVA database SSU Ref NR 99 release 138 [8].

For analysis of clinical outcomes, patients were stratified into higher diversity and lower diversity according to the median microbial diversity value obtained. Diversity was also analyzed as a continuous variable. Results were adjusted for multiple comparisons using the Tukey procedure.

Patients were treated with Ixazomib (4 mg, d 1, 8, and 15), thalidomide (100 mg/d), and dexamethasone (40 mg once/week) with dose reduction of thalidomide and dexamethasone in patients aged ≥75 years. After eight cycles of therapy, patients received ixazomib maintenance therapy (4 mg, days 1, 8, 15 of a 28 cycle and 3 mg in patients aged ≥75 years) for one year [9]. FISH analysis was performed on CD138 selected bone marrow plasma cells. The cutoff level for positivity was 10% for *t*(4;14), and for *t*(14;16), 20% for amp1q21, and 60% for del(17p), respectively. Cytogenetic high risk was defined as *t*(4;14) ± del 17p, ± amp1q21. Progression‐free survival (PFS) and overall survival (OS) was estimated according to Kaplan‐Meier, and differences were evaluated by the log‐rank test. Response rates were compared using Fisher's exact test. Median follow‐up was 27.2 months. The study has been approved by the relevant ethical committees and is registered under NCT02410694 (clinical.trials.gov).

## 3 RESULTS

**FIGURE 1:**
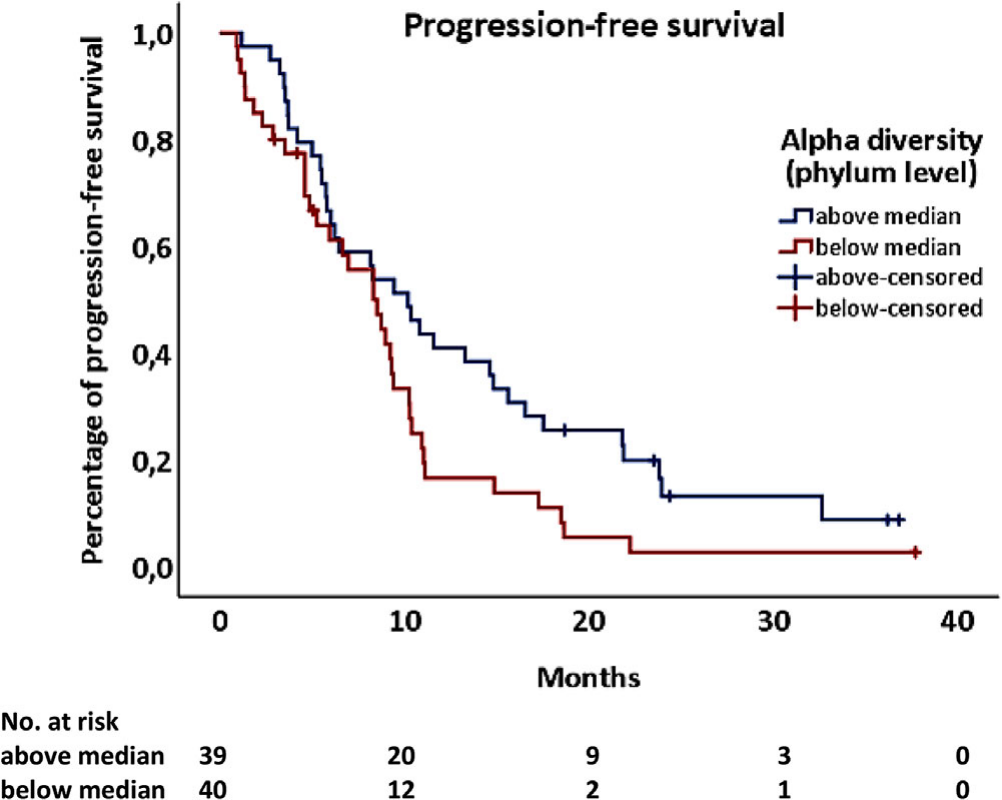
Patients with a high alpha diversity (above median) at the phylum level of the microbiome had a significantly longer PFS (10.2 vs 8.5 months, *P* = .040)

**FIGURE 2:**
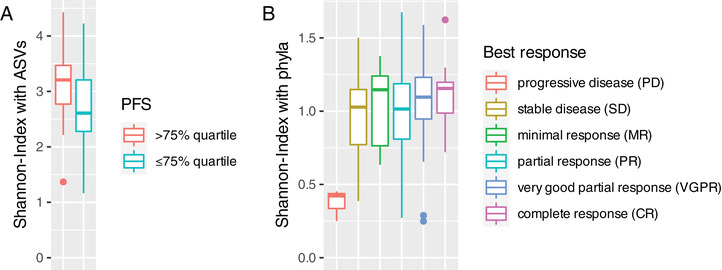
(A) Patients with particularly long PFS (>75% quartile vs ≤75% quartile) had a significantly higher alpha diversity (Shannon index on the ASV level 3.2 vs 2.7, *t*‐test, *P* < .05). (B) Patients with progressive disease showed a significantly lower alpha diversity compared to any of the other outcomes (Shannon index at the phylum level, ANOVA with Tukey's post hoc test, *P* < .05)

The composition of human buccal mucosa microbiome samples was dominated by Streptococcus, Pasteurellaceae, and Veillonella species, which is consistent with previously published studies (http://www.homd.org) [10]. No major differences were observed in the microbiome between the entire group of patients and the general population. However, patients with a high alpha diversity (above median) at the phylum level of the microbiome had a significantly longer PFS (10.2 vs 8.5 months, *P* = .040, Figure 1). Similarly, when the microbiome was analyzed in patients with very long PFS (>75% quartile vs <75% quartile) an even higher diversity of the microbiome was noted (Shannon index on the ASV level 3.2 vs 2.7, *t*‐test, *P* < .05 Figure 2A). Of note, the spilt of patients in quartiles of the diversity of the microbiome showed a constant trend (Supplementary Figure 1). Furthermore, in the small subgroup of patients with progressive disease, a significantly reduced alpha diversity of the microbiome was observed compared to all others (Shannon index on phylum level, ANOVA with Tukey's post hoc test, *P* < .05, Figure 2B). The diversity on the ASV and genus level showed the same trends, but were not statistically significant. Further analysis did not reveal statistically significant differences in the diversity of the microbiome between patient groups split by the median of microbiome diversity (Supplementary Table 1), patients of different gender, age (<75 versus ≥75 years of age), with two or more than two prior lines of therapy, or those with more or less than the median time from diagnosis to enrollment (3.8 years) in this study. Similarly, no differences were noted in the baseline composition of the buccal microbiome between patients with different levels of response (from CR to MR), high‐risk or standard‐risk cytogenetics, shorter or longer median PFS (8.8 months) and shorter or longer median OS (42 months).

## 4 DISCUSSION

This study is, to our knowledge, the first aimed at investigating possible relationships between the composition of the oral microbiome and outcome in uniformly treated patients with relapsed/refractory multiple myeloma. We found a significant association between a reduced alpha diversity of the oral microbiome at the phylum level and shorter PFS; an observation that was particularly pronounced in patients with very short PFS, and in those with progressive disease. However, we did not observe strong enrichments or depletions of specific bacterial species in patients with shorter PFS, nor depletions of specific taxa in those with progressive disease. This may be attributable to the Anna Karenina principle of microbiome dysbiosis [11], which implies a greater variability in dysbiotic individuals than in those with a eubiotic microbiome. We acknowledge that the number of investigated patients is limited, which may in part explain the lack of qualitative differences in the oral microbiome between our patients and the general population. In rheumatoid arthritis, a link between oral and gut microbiota has already been established [12]; in cancer, most studies have focused on the gut microbiome solely. A recent study in patients subjected to allogeneic transplantation including 111 patients with multiple myeloma showed loss of diversity in the fecal microbiota and outgrowth of single taxa across the entire patient cohort [13]. Diversity significantly decreased shortly after transplantation, and reduced diversity at pre‐transplant was associated with higher mortality. In a cohort of conventionally treated myeloma patients, Zhang et al. found a significantly lower diversity of the gastrointestinal microbiome, as measured by the Shannon index, compared to the general population [4]. Furthermore, they showed marked differences in the abundance of several bacterial taxa. At the phylum level, a higher abundance of Proteobacteria and lower abundance of Actinobacteria were identified, while at the genus level the proportion of Bacteroides, Faecalibacterium, and Roseburia was significantly higher in multiple myeloma. This was also true for *Pseudomonas aeruginosa* and Faecalibacterium; interestingly, a correlation between the abundance of the latter and the ISS stage was also noted. Another report on 34 multiple myeloma patients, who had been tested for MRD (minimal residual disease) status after induction therapy including autologous transplantation in 41%, showed a higher relative abundance of *Eubacterium hallii* in the 16 MRD^neg^ compared to the 18 MRD^pos^ patients, though no difference in alpha diversity was observed between the two groups [5]. The authors argued that *Faecalibacterium prausnitzii* and *Eubacterium hallii* are common butyrate‐producing commensals. Butyrate and other short‐chain fatty acids, such as propionate and acetate, are biologically active metabolites formed during microbial fermentation of dietary and host‐derived carbohydrates, which supply the host with energy, and modulate immunity via exertion of anti‐inflammatory functions. Butyrate inhibits IL‐17 production via downregulation of Th 17 cells [14]. Calcinotto et al provided compelling evidence for the regulatory role of the microbiome via modulation of TH 17 cell function in transgenic Vk:MYC transgenic mice [1]. This genetically engineered mouse model is based on the dysregulation of myc and is considered a faithful model of untreated multiple myeloma [15]. Specific bacteria of the intestinal tract such as Prevotella, *Citrobacter rodentium*, *Schaedler flora, E. coli* 0157, and SFB (segmented filamentous bacteria), if present in abundance, promote the differentiation and activation of Th17 cells of the intestinal mucosa [15]. After activation, these cells migrate to the bone marrow where they stimulate progression of multiple myeloma. Treating these mice with antibiotics reduced myeloma progression and increased survival [15], emphasizing the delicate interplay between gastrointestinal microbiota and tumor progression. In humans, more research and information on the role of the microbiome and its modulation by myeloma drugs such as proteasome inhibitors and IMiDs (immunomodulatory drugs), both of which may exert gastrointestinal toxicity, is needed before we eventually will be able to modulate progression of multiple myeloma by modification of the microbiome.

In conclusion, our data show a high alpha diversity of the oral microbiome in patients with longer PFS, and particularly in patients with very long PFS. Furthermore, in the small subgroup of patients with progressive disease, a significantly reduced alpha diversity of the microbiome was noted. Further studies are needed to evaluate whether these imbalances may improve with effective therapy and whether modulation of the microbiome affects the course of the disease.

## CONFLICT OF INTEREST

HL: Research Funding: Takeda, Amgen; Speaker's Bureau: Takeda, Amgen, Janssen, BMS, Celgene; Consultancy Fees: PharmaMar; SK: Consultancy Fees: Takeda; EG: Honoraria: Takeda, Janssen, Amgen, BMS; Advisory Board: Takeda, Janssen, Amgen, Novartis; AP: Honoraria, Advisory Board: Takeda, Celgene; KW: Honoraria: Novartis, Janssen, Celgene, Amgen, Onyx, Takeda, BMS; Consultancy Fees: Janssen, Celgene,

Amgen, BMS, Takeda, Onyx; RG: Research Funding: Roche, Celgene, Takeda, Novartis; Personal Fees: Roche, Takeda, BMS, Amgen; HE: Speaker's Bureau, Advisory Board: Celgene, Janssen, Amgen, BMS, Novartis; Consultancy Fees, Honoraria: Celgene, Janssen, BMS, Amgen; WW: Research Funding: Amgen, BMS, Celgene, Janssen, Novartis, Roche, Takeda; Advisory Board/Consultancy fees: Amgen, BMS, Celgene, Janssen, Novartis, Pfizer, Roche, Sandoz, Takeda; The remaining authors declare no competing financial interests.

## Supporting information

Supplementary Table 1. Patient characteristics split by the median of the microbiome diversity (Shannon index at phylum level)

Supplementary Figure 1. Microbiome diversity split by quartiles showing a constant trend over the various categories

## Data Availability

Sequencing data will be submitted to the portal of the U.S. National Library of Medicine, National Center for Biotechnology information and will be made publicly available.
