# Supplementary material for: Reduced alpha diversity of the oral microbiome correlates with short progression‐free survival in patients with relapsed/refractory multiple myeloma treated with ixazomib‐based therapy (AGMT MM 1, phase II trial)
Source: EJHaem. 2020 Nov 8;2(1):99–103. doi: 10.1002/jha2.130 (PMC9176146; doi:10.1002/jha2.130)
Supplement: Supplementary file 1 — Supplementary Table 1. Patient characteristics split by the median of the microbiome diversity (Shannon index at phylum level) [file JHA2-2-99-s002.docx]

# Supplementary Table 1. Patient characteristics split by the median of the

# microbiome diversity (Shannon index at phylum level)

| **~~Patient characteristics split by median of microbiome diversity (Shannon index at phylum level~~)** | | |
| --- | --- | --- |
| **All patients (n=79)** | **Below median of microbiome diversity (n=40)** | **Above median of microbiome diversity (n=39)** |
| **Age,** years (range) | 69.6 (45.9-84.4) | 66.9 (50.6-83.6) |
| **Gender**, male/female | 21 (52.5%) / 19 (47.5%) | 18 (46.2%) / 21 (53.8%) |
| **ISS Stage**: I / II / III | 16 (40%) / 13 (32.5%) / 11 (27.5%) | 16 (41%) / 14 (35.9%) / 9 (23.1%) |
| **ECOG** 0-1/2 | 37 (92.5%) / 3 (7.5%) | 38 (97.4%) / 1 (2.6%) |
| **Cytogenetics** | | |
| t(4;14) and/or del(17p) | 9 (32.1%) | 6 (23.1%) |
| Gain of 1q21 | 13 (52%) | 15 (57.7%) |
| No aberration | 9 (40.9%) | 10 (38.5%) |
| **MM Isotype** | | |
| IgG/IgA/light chain | 21 (52.5%) / 10 (25%) / 9 (22.5%) | 23 (59%) / 8 (20.5%) / 8 (20.5%) |
| **Prior treatment lines** | | |
| 1-2 / 3-4 / ≥5 | 27 (67.5%) / 8 (20%) / 5 (12.5%) | 32 (82.1%) / 5 (12.8%) / 2 (5.1%) |
| ASCT | 23 (57.5%) | 27 (69.2%) |
| **Months since start of 1^st^ line TX** | 47.6 (10.2-230.0) | 45.8 (11.3-152.0) |

Figure Legends

Supplementary Figure 1. Microbiome diversity split by quartiles showing a constant trend over the various categories
