# Supplementary figures and images for: Reduced alpha diversity of the oral microbiome correlates with short progression‐free survival in patients with relapsed/refractory multiple myeloma treated with ixazomib‐based therapy (AGMT MM 1, phase II trial)
Source: EJHaem. 2020 Nov 8;2(1):99–103. doi: 10.1002/jha2.130 (PMC9176146; doi:10.1002/jha2.130)

Shannon-Index with ASVs

4  
3  
2  
1  
0

Quartiles

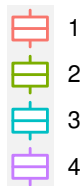

Supplement: Supplementary file 2 — Supplementary Figure 1. Microbiome diversity split by quartiles showing a constant trend over the various categories [file JHA2-2-99-s001.pdf]
